# A systematic review of oculomotor deficits associated with acute and chronic cannabis use

**DOI:** 10.1111/adb.13359

**Published:** 2023-12-19

**Authors:** Brooke Manning, Luke A. Downey, Andrea Narayan, Amie C. Hayley

**Affiliations:** ^1^ Centre for Mental Health and Brain Science, School of Health Sciences Swinburne University of Technology Hawthorn Victoria Australia; ^2^ International Council for Alcohol, Drugs and Traffic Safety (ICADTS) Rotterdam Netherlands; ^3^ Institute for Breathing and Sleep Austin Hospital Melbourne Victoria Australia

**Keywords:** cannabis, driving, eye movement, oculomotor, saccadic, THC

## Abstract

Driving is a critical everyday task necessitating the rapid and seamless integration of dynamic visually derived information to guide neurobehaviour. Biological markers are frequently employed to detect Δ9‐tetrahydrocannabinol (THC) consumption among drivers during roadside tests, despite not necessarily indicating impairment. Characterising THC‐specific alterations to oculomotor behaviour may offer a more sensitive measure for indexing drug‐related impairment, necessitating discrimination between acute THC effects, chronic use and potential tolerance effects. The present review aims to synthesise current evidence on the acute and chronic effects of THC on driving‐relevant oculomotor behaviour. The review was prospectively registered (10.17605/OSF.IO/A4H9W), and Preferred Reporting Items for Systematic Reviews and Meta‐Analyses (PRISMA) guidelines informed reporting standards. Overall, 20 included articles comprising 12 experimental acute dosing trials, 5 cross‐sectional chronic use studies and 3 roadside epidemiological studies examined the effects of cannabis/THC on oculomotor parameters including saccadic activity gaze behaviour, nystagmus, smooth pursuit and eyelid/blink characteristics. Acute THC consumption selectively impacts oculomotor control, notably increasing saccadic latency and inaccuracy and impairing inhibitory control. Chronic cannabis users, especially those with early age of use onset, display enduring oculomotor deficits that affect visual scanning efficiency. The presence of eyelid tremors appears to be a reliable indicator of cannabis consumption while remaining distinct from direct impairment associated with visual attention and motor control. Cannabis selectively influences oculomotor activity relevant to driving, highlighting the role of cannabinoid systems in these processes. Defining cannabis/THC‐specific changes in oculomotor control may enhance the precision of roadside impairment assessments and vehicle safety systems to detect drug‐related impairment and assess driving fitness.

## 1 INTRODUCTION

The global increasing availability of cannabis for recreational and medicinal purposes raises concerns regarding its impact on safety‐sensitive activities such as driving.^1^, ^2^ Extant research indicates that acute Δ9‐tetrahydrocannabinol (THC) use disrupts neurocognition, augmenting dopamine release and increasing the activation of cannabinoid type 1 (CB1) receptors in key areas of the brain essential for cognitive functioning and motor control and altering oculomotor processes critical to the selection and uptake of visual information.^3^, ^4^, ^5^ Driving‐related behavioural impairments may be quantified through eye movement analysis under various intoxication profiles.^6^ Roadside tests commonly use oculomotor measures such as smooth pursuit and nystagmus and vehicle safety systems increasingly monitor ocular markers including saccadic activity and gaze behaviour to detect drug‐induced impairment.^7^, ^8^, ^9^ Presently, these methods lack the requisite specificity to differentiate cannabis intoxication from other associated states that may negatively impact driving (such as fatigue). Moreover, current strategies for roadside detection of cannabis use rely on positive THC biomarker tests, which do not inherently indicate impairment. As such, there is a critical need to define cannabis/THC specific changes to oculomotor control, particularly along the broad spectrum of consumption patterns, to distinguish between acute THC intoxication, potential long‐term impacts of chronic use and the possible development of tolerance to impairment effects in more frequent cannabis users.

Involuntary rapid eye movements (REMs) occurring between visual fixations, termed saccadic eye movements, have demonstrated the potential to act as a practical biomarker of driving performance.^10^ Saccadic eye movements are often measured by their amplitude, latency, accuracy and peak velocity (SPV), with alterations to these indices often indicating decreased attentional capacity or increased physiological signs of drowsiness.^11^ Reductions in SPV have been observed to mirror overall alertness and exhibit sensitivity to the effects of drugs and alcohol.^12^ Consequently, heightened saccadic activity has been linked to greater lane deviation, which is considered a primary predictor of crash risk.^13^ Similarly, those experiencing nystagmus, a condition characterised by rhythmic involuntary eye movements and disrupted smooth pursuit, demonstrate a compromised ability to visually scan their driving environment.^14^ Considering recent studies indicating the involvement of the cannabinergic system in the high‐level control of saccadic activity and involuntary eye movement, such measures may provide an objective means of quantifying and indexing motor and cognitive effects after cannabis use.^15^

Gaze behaviour, which evaluates how attention is spatially and temporally distributed during interspersed fixations occurring between eye movements,^16^ exhibits a useful behavioural output for assessing visual scanning, an essential component for safe driving.^11^ Prior research has noted prolonged fixation duration among long‐term cannabis users, suggesting that cannabis use may correspond with dysfunctional attentional shifts and delayed information processing.^17^ As driving is a highly visual task, requiring rapid uptake and processing of visual information, these mechanistic deficits may impede performance in tasks that necessitate this type of high‐level processing. Earlier studies have established a link between measurable eyelid characteristics, such as frequency, amplitude, velocity and duration of eyelid closure and performance on driving simulations.^14^ Cannabis use has been shown to induce eyelid tremors and a drooping eyelid effect, which is often reported among cannabis users.^7^, ^8^ Consequently, tracking of gaze and eyelid markers may further assist in the indexation of cannabis‐related alterations to visual attention, specifically in the context of driving.

Epidemiological evidence suggests that cannabis use can moderately increase crash involvement^18^ due to the disruption of oculomotor control processes and reduction of sustained attention abilities^19^ necessary for driving, emphasising the need to clarify the measurable effects of cannabis on oculomotor activity relevant to driving. This review aims to synthesise the current clinical, experimental and observational evidence on the acute and chronic effects of cannabis/THC on oculomotor behaviour and subsequent impact on functional attention relevant to driving.

## 2 METHOD

### 2.1 Protocol and registration

The current review was prospectively registered (Open Science Framework, registration DOI 10.17605/OSF.IO/A4H9W), and the Preferred Reporting Items for Systematic Reviews and Meta‐Analyses (PRISMA) guidelines were used to guide reporting standards.^20^

### 2.2 Eligibility criteria

Studies fulfilling the following criteria were eligible for inclusion as follows: (1) experimental or observational studies involving cannabis/THC consumption; (2) assessment of a control, comparison or baseline group; and (3) measurement of oculomotor outcomes relevant to driving, including saccadic activity (fixation, saccade), gaze behaviour (smooth‐entropy: stationary, transition), eyelid characteristics (closure, droopiness, or tremors), blink behaviour (velocity, duration, or frequency) or nystagmus (horizontal or vertical gaze). Articles were excluded if they: (1) had no full‐text available, (2) were nonhuman studies, (3) involved primarily nonadult populations, (4) involved administration or use of synthetic cannabinoids or phytocannabinoids only, (5) were an observational study with only self‐reported cannabis use as a confirmatory measure or (6) measured changes in eye movement associated with REM during sleep, schizophrenia or cannabis‐induced psychosis.

### 2.3 Database selection and search strategy

A search string of [(cannabi* OR marijuana OR thc OR tetrahydrocannabinol) AND (ocul* OR visuomotor OR saccad* OR “eye movement*” OR gaze* or fixation* OR blink* OR eyelid*)] was formulated with assistance from a research librarian to identify scholarly peer‐reviewed journal articles. Two reviewers (BM and AN) completed a database search (EBSCOhost, PsycNet, PubMed, Scopus and Web of Science) screening articles published up to November 2022 independently and in duplicate. Inaccessible full‐text articles were requested directly from authors. Consensus was met through discussion or third reviewer adjudication (AH). A forward‐search was also completed on the 17th of July 2023 to ensure that the most updated source data were included for final review. This was conducted independently by BM and AN using the outlined parameters per database and study type. The search range examined studies published from December 2022 to July 2023, yielding one additional study eligible for review.^21^ The full search criteria for this and resultant publications per database are available upon request.

### 2.4 Risk of bias (RoB) and quality assessment

Two independent reviewers (BM, AN) assessed included articles for RoB, with consensus achieved without requiring a third‐party adjudicator. Randomised crossover studies were assessed using the Cochrane RoB 2 tool and revised additional considerations for crossover trials.^22^, ^23^ Observational studies were assessed using the Cochrane RoB in Non‐Randomised Studies of Interventions (ROBINS‐I) guidance tool.^24^ Epidemiological studies were assessed for bias using the Critical Analysis of Skills Programme checklist for case control studies.^25^

### 2.5 Data collection and synthesis

Demographic characteristics were extracted from each article, including number of participants, sex, age,and cannabis use history. Article results were collated and organised based on primary outcome of interest before being stratified further by study design. In instances where measures were assessed at multiple timepoints within experimental studies and results varied between timepoints, results are presented for each when available. Due to the significant heterogeneity among the studies included in our review, effect estimates and meta‐analysis were not performed.

## 3 RESULTS

### 3.1 Article identification and selection

**FIGURE 1:**
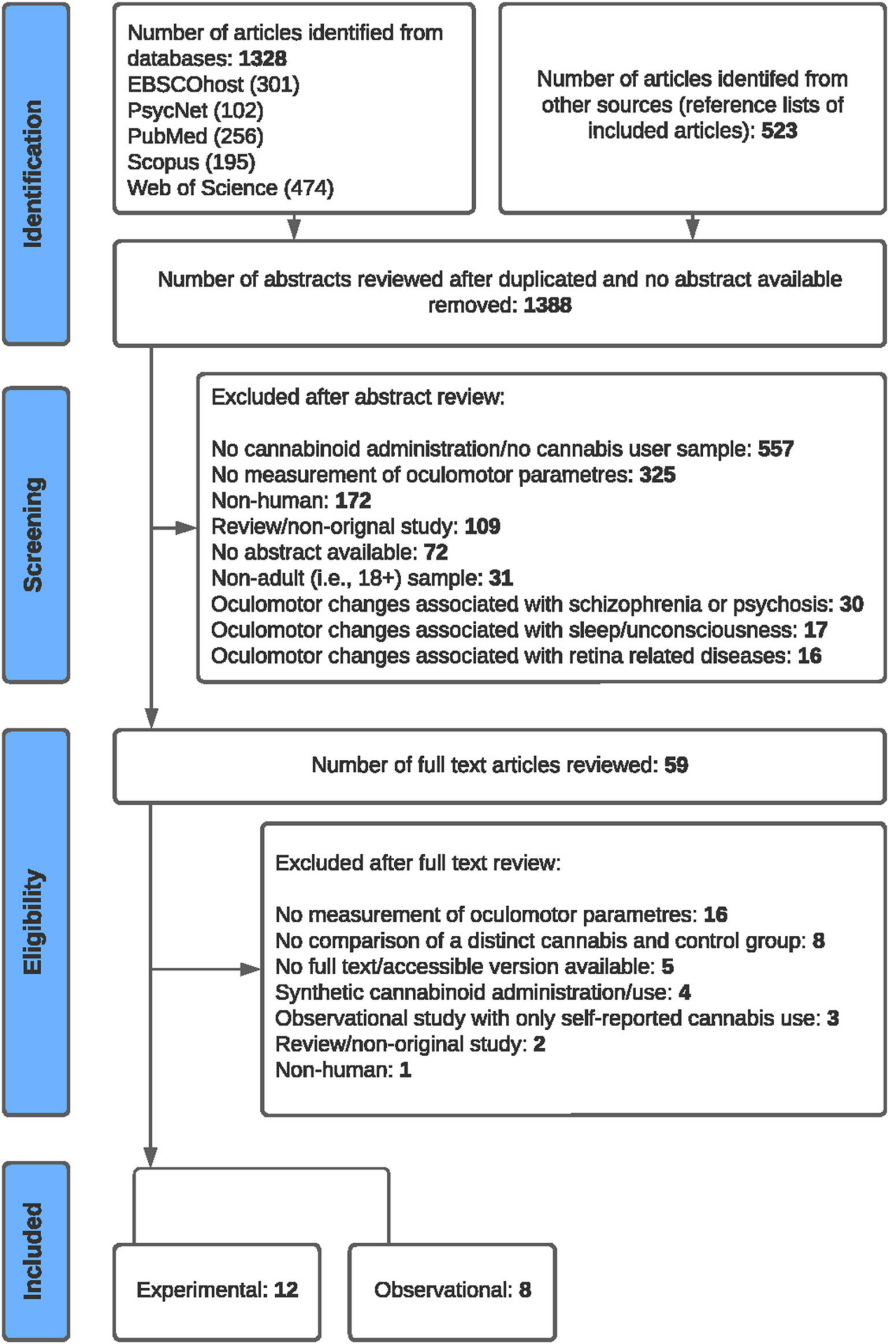
Preferred Reporting Items for Systematic Reviews and Meta‐Analyses (PRISMA) flow diagram.

After removing duplicates, 1388 citations underwent screening by two reviewers independently and in replicate. Of these, 59 citations were considered potentially eligible and underwent full‐text assessments for inclusion. Twenty studies ultimately met eligibility criteria, comprising twelve experimental (nine randomised controlled trials and three nonrandomised/pilot studies) and eight observational studies (five cross‐sectional and three epidemiological studies). The adapted PRISMA Flow Diagram (Figure 1) illustrates the selection process for inclusion in the review, with primary reasons for exclusion at full‐text assessment noted.

### 3.2 Participants

Demographic characteristics of included studies are shown in Table A1. A total of 2699 participants were included in the reviewed studies, ranging from a minimum sample size of 10 participants to a maximum sample size of 843 participants. Two articles^17^, ^26^ used an identical sample for their reports and are counted once in the demographic data. In experimental and observational cross‐sectional studies, ages ranged from 18 to 65 years, with two studies reporting an average age of ≥ 30.^21^, ^27^ In a single epidemiological study, ages ranged from 15 to 59 years.^7^ A male‐only sample was utilised in five studies^28^, ^29^, ^30^, ^31^, ^32^ with a further nine studies reporting a higher proportion of male than female subjects.^7^, ^17^, ^26^, ^27^, ^33^, ^34^, ^35^, ^36^, ^37^ One study involved a sample with an equal ratio of male to female participants^38^ and three studies' samples included more females than males.^15^, ^21^, ^39^ Two epidemiological studies did not report participant sex or age.^8^, ^40^ Studies were predominantly conducted in the United States^7^, ^8^, ^27^, ^28^, ^31^, ^37^ and the Netherlands.^29^, ^30^, ^32^, ^33^, ^38^ The remaining studies originated from Germany,^15^, ^17^, ^26^, ^35^ Australia^21^, ^34^, ^36^ and Canada.^39^, ^40^

### 3.3 Cannabis consumption

#### 3.3.1 Cannabis administration

Throughout included experimental studies, THC was predominantly administered via inhalation methods, comprising of THC only cigarettes,^28^, ^31^, ^33^, ^34^, ^36^, ^38^, ^39^ or THC in a vaporised form.^29^, ^30^, ^32^ Two experimental studies administered THC via an oral solution.^15^, ^21^

#### 3.3.2 Ongoing cannabis use

Five observational cross‐sectional studies included participants who met diagnostic criteria for cannabis use disorder (CUD) and tested positive for the presence of THC in biological confirmatory measures during testing sessions. Of these, three studies instructed participants to remain abstinent from cannabis use for approximately 24 h prior to testing.^17^, ^26^, ^35^ Reported cannabis consumption in ongoing cannabis user groups included those who solely smoked cannabis cigarettes^17^, ^26^, ^35^ or had no sole specified route of administration.^27^, ^37^ The remaining three epidemiological studies involved unknown administration routes as they comprised of populations who were stopped for suspected DUI.^7^, ^8^, ^40^

#### 3.3.3 Dosage

Most experimental studies administered doses of THC only; ranging from 5.9 mg^38^ to 89.49 mg^39^ within a single session. In three studies, THC dosages were calculated for each individual participant based on their body weight, ranging from 100 μg/kg^38^ to 400 μg/kg.^33^ Another three studies utilised consecutive doses of THC ranging from 12 mg THC^29^, ^30^ to 20 mg THC^32^ within a single study session. A single experimental study administered a combined cannabinoid treatment of 5 mg THC and 100 mg cannabidiol.^21^ THC dosages for several studies are unknown due to the inclusion of populations who were ongoing cannabis users^17^, ^26^, ^27^, ^35^, ^37^ or were found to test positive for THC at the roadside^7^, ^8^, ^40^ and, by design, were not experimentally administered quantifiable THC or cannabis dosages during study sessions.

### 3.4 Measures

Research‐grade eye tracking systems were primarily utilised to capture pupil and corneal reflections via an infrared head‐mounted device to measure saccadic activity, gaze behaviour and blink characteristics. The EyeLink device^17^, ^26^, ^35^ was the most used eyetracker, offering the highest reported sampling rate of 250 Hz. Other devices, ranked in descending order of sampling rate, included the Eyetracker,^15^ Eye Performance System 100,^28^ 4000SU Eye‐Tracker,^38^ Eye Point of Regard,^31^ MiraMetrix S2,^37^ SmartEye Pro 8.0^39^ and SensoMotoric Instruments eyetracker with the lowest sampling rate of 50 Hz.^21^ Several studies measure oculomotor outcomes using electrodes placed next to the lateral canthi of the right eye^30^ or simultaneously on the forehead that were stimulated sinusoidally (frequencies ranging 0.3–1.1 Hz) with amplitudes between 20° and 22.5° on each side.^29^, ^32^

Nystagmus and eyelid characteristics were measured by individuals trained to perform Standardised Field Sobriety Tests (SFST)^27^, ^33^, ^34^, ^36^ or the Drug Evaluation and Classification (DEC) Programme.^7^, ^8^, ^40^ Assessors looked for ‘clues’ in each eye indicating a lack of smooth pursuit, distinct nystagmus at maximum deviation and nystagmus onset before 45°, with four clues across both eyes indicating the presence of horizontal gaze nystagmus (HGN). In determining vertical gaze nystagmus (VGN) impairment, assessors identified the presence or absence of nystagmus at maximum deviation in an upward vertical gaze. Eyelid tremors were also often noted by assessors during SFST and DEC tests.

### 3.5 Effect of cannabinoids on oculomotor parameters

#### 3.5.1 Saccadic activity

Eight studies evaluated various aspects of saccadic activity, including saccade velocity, amplitude, latency and accuracy (Table A1). Of these, five experimental studies administered acute THC doses: a single 10 mg dose,^15^ an average of 62.72 mg dose^39^ and consecutive THC doses of 2, 4 and 6 mg,^29^, ^30^ with one study also administering a fourth consecutive THC dose of 8 mg.^32^ Saccadic latency, referring to the time taken prior to initiation of eye movement, increased post‐THC administration relative to placebo.^32^ This increase in latency was also observed in visually guided tasks (i.e. eye movements made voluntarily towards a visual stimulus), relative to baseline measurements,^15^ although this finding was not consistently replicated.^29^ Acute THC administration was also associated with increased occurrences of anticipatory memory‐guided saccades, involving the eyes moving towards a previously visualised location, even before the target appeared.^15^

Following acute THC administration, saccadic accuracy (i.e. the ability to accurately reach a target location) showed a significant decrease compared with placebo.^29^ Deficits in saccadic accuracy were also observed during a memory‐guided task; however, were not evident during a visually guided task.^15^ Finally, amplitude constants, which measure the extent of eye movement during saccades, did not show significant differences in either visually guided or memory‐guided tasks relative to baseline measurements.^15^

Saccadic activity was assessed in three observational studies involving ongoing cannabis users who met diagnostic criteria for CUD.^26^, ^35^, ^37^ CUD groups exhibited significantly increased saccade amplitudes,^35^ antisaccade initial amplitudes and memory‐guided saccade amplitudes at 3° eccentricity.^26^ Although similar variations in both initial prosaccade and memory‐guided saccade amplitudes at 6° eccentricity were not apparent.^26^ Results indicate that prosaccade and antisaccade latencies may be increased in chronic cannabis users, with increases shown in initial latencies during overlap trials compared with gap trials.^26^ A significant increase in antisaccade errors was also reported in a CUD group relative to a control group^37^; however, this was not replicated in prosaccade tasks, with no differences in the number of errors made between CUD and control groups.^26^, ^37^ No differences in SPV were reported following THC consumption relative to placebo,^29^, ^30^, ^32^ baseline,^15^ or control group.^35^

#### 3.5.2 Gaze behaviour

Gaze behaviour, including visual search and fixation, was assessed in six studies (Table A2). Two experimental trials investigated acute THC dosages of 100^38^ and 200 μg/kg^31^ via a cigarette, with no differences observed in visual search characteristics or gaze behaviour in THC conditions relative to placebo. One experimental study showed significantly longer fixation duration, following acute oral administration of an oil solution containing 5 mg THC, compared with placebo.^21^ An additional pilot study noted an increase in gaze pitch angle following THC cigarette smoking (average dose 62.72 mg) compared with baseline; however, no other differences were apparent in gaze or fixation outcomes.^39^ Two observational studies of ongoing cannabis users who met diagnostic criteria for CUD showed significant increases in the average number of fixations per item,^35^ fixation duration and number of visual regressions,^17^ compared with matched control groups. However, prior research did not yield comparable results, with no differences in average fixation duration between CUD and control groups observed.^35^

#### 3.5.3 Nystagmus

Nystagmus was evaluated in seven studies (Table A2) by the presence or absence of HGN or VGN. No impairment in HGN was observed during DEC tests in drives suspected of DUI who tested positive for THC, compared with those who did not test positive for alcohol or other drugs.^7^, ^8^, ^40^ Conversely, participants with ongoing cannabis use who met diagnostic criteria for CUD exhibited a significant increase in HGN impairment during SFST relative to matched drug‐free controls.^27^ Another study observed a trend towards increased HGN impairment during SFST after acute inhalation of 400 μ/kg THC via, relative to baseline.^33^ Two randomised controlled trials showed significant increases in the impairment of HGN during SFST in those who smoked a cigarette (1.776 g) containing 2.93% THC at both 5 and 105 min after administration^36^ and cigarettes (0.81 g/1.78 g) containing either 1.8% or 3% THC at 50 min after administration.^34^ Three studies examined VGN presence and found no differences between drug cases and nondrug cases^7^ or acute THC administration versus placebo.^34^, ^36^

#### 3.5.4 Smooth pursuit

The effect of THC on smooth pursuit was examined in four experimental studies (Table A2). Consecutive THC doses of 2, 4 and 6 mg^29^, ^30^; a fourth consecutive THC dose of 8 mg^32^; and single acute THC doses of 15.6 or 25.1 mg^28^ were compared with placebo. Two randomised controlled trials showed no significant differences in smooth pursuit between THC and placebo groups.^29^, ^32^ In contrast, two studies revealed smooth pursuit alterations, marked by a significant decrease in the percentage of time of which eye movements were in smooth pursuit^30^ and a decrease in pursuit speeds overall and across time in the 25.1 mg THC condition.^28^

#### 3.5.5 Eyelid characteristics

The effect of THC on eyelid characteristics including blink duration, blink frequency, eyelid closure and eyelid tremors was assessed in five studies (Table A2). Three epidemiological studies involving participants stopped at the roadside for suspected DUI that suggest that cannabis use may be linked to the occurrence of eyelid tremors, with 57%^40^ and 58%^7^ of cannabis user cases demonstrating eyelid tremors and a significant increase in eyelid tremors among drivers who tested positive for THC compared with those who had not used any drugs.^8^ In a single experimental study, blink duration was significantly decreased following acute oral administration of an oil solution containing 5 mg THC, compared with placebo.^21^ An experimental pilot study reported no differences in blink duration, blink frequency or eyelid opening in those who smoked a cigarette containing THC (mean 62.72 mg) compared with baseline.^39^

### 3.6 RoB assessment

Twenty studies were evaluated for RoB, with *robvis* ^41^ used to illustrate ‘traffic light’ plots of domain level judgements and weighted bar plots showing the distribution of RoB judgements within each domain.

#### 3.6.1 RoB assessment outcomes for randomised crossover studies

**FIGURE 2:**
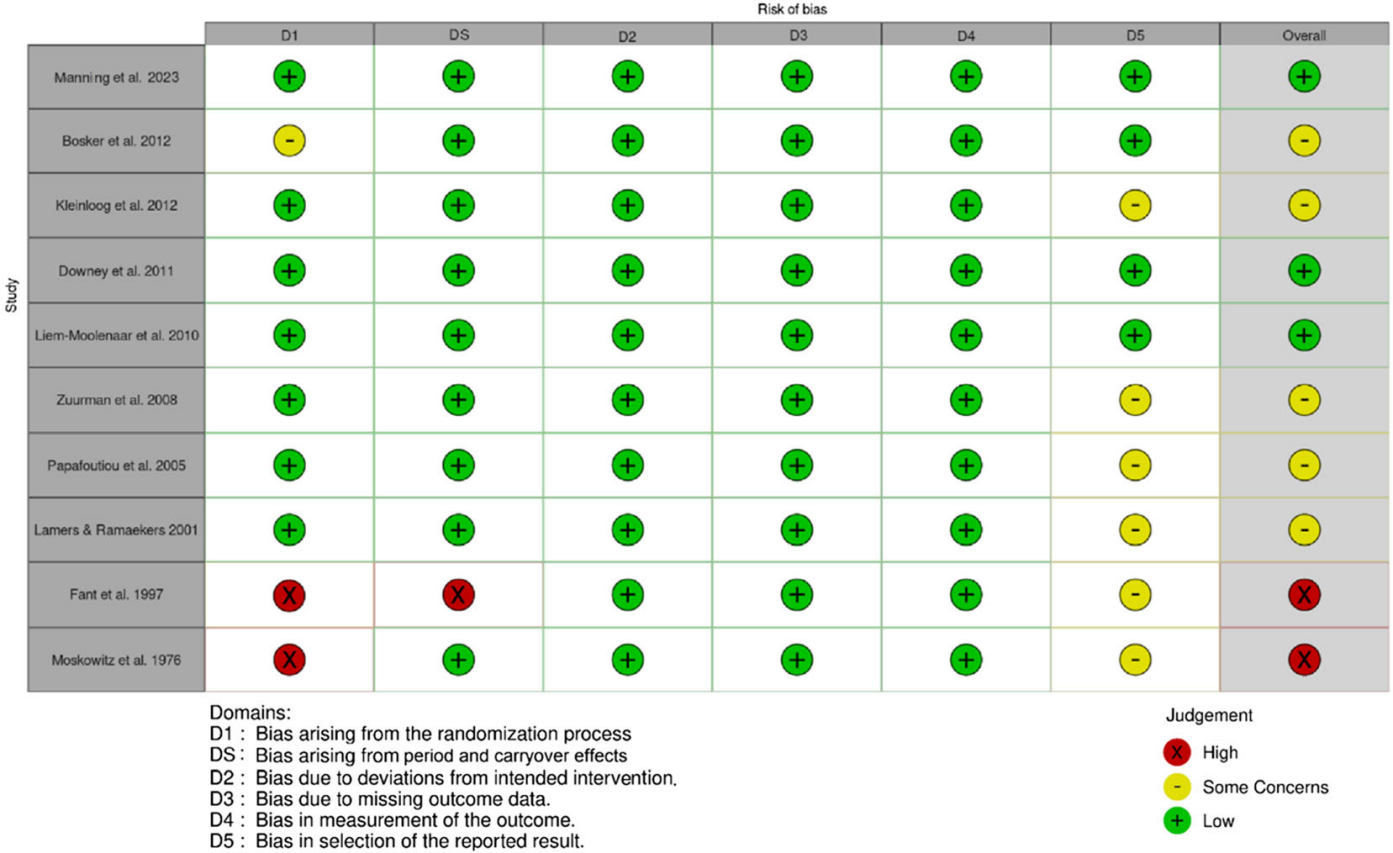
Domain and overall Cochrane Risk of Bias (RoB2) judgements for included randomised crossover studies (reverse chronological order).

**FIGURE 3:**
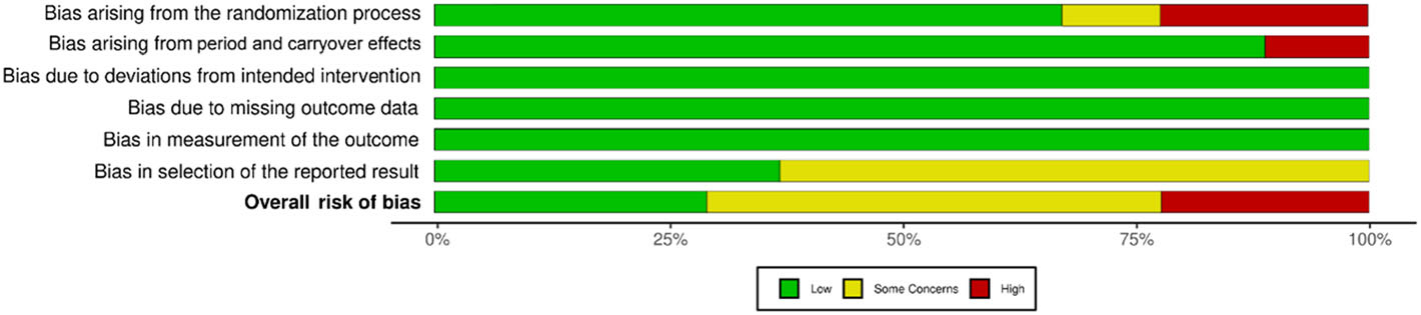
Domain and overall Cochrane Risk of Bias (RoB 2) judgement summary plot for included randomised crossover studies.

Randomised crossover studies ranged from a low to high RoB as shown in Figures 2 and 3. Most randomised crossover studies had some concerns regarding selection bias, with a statistical analysis plan unavailable in 60% of studies. One study had some concerns due to the unavailability of reported information on whether the allocation sequence was sufficiently concealed.^33^ Two studies had a high RoB, with one not meeting standards for appropriate randomisation due unavailable randomisation information^31^ and the second lacking allocation sequence concealment and sufficient time for potential carryover effects to have subsided.^28^ Overall, 20% of studies had a high RoB, 50% of studies had some concerns and 30% of studies were deemed at low RoB.

#### 3.6.2 RoB assessment outcomes for nonrandomised studies

**FIGURE 4:**
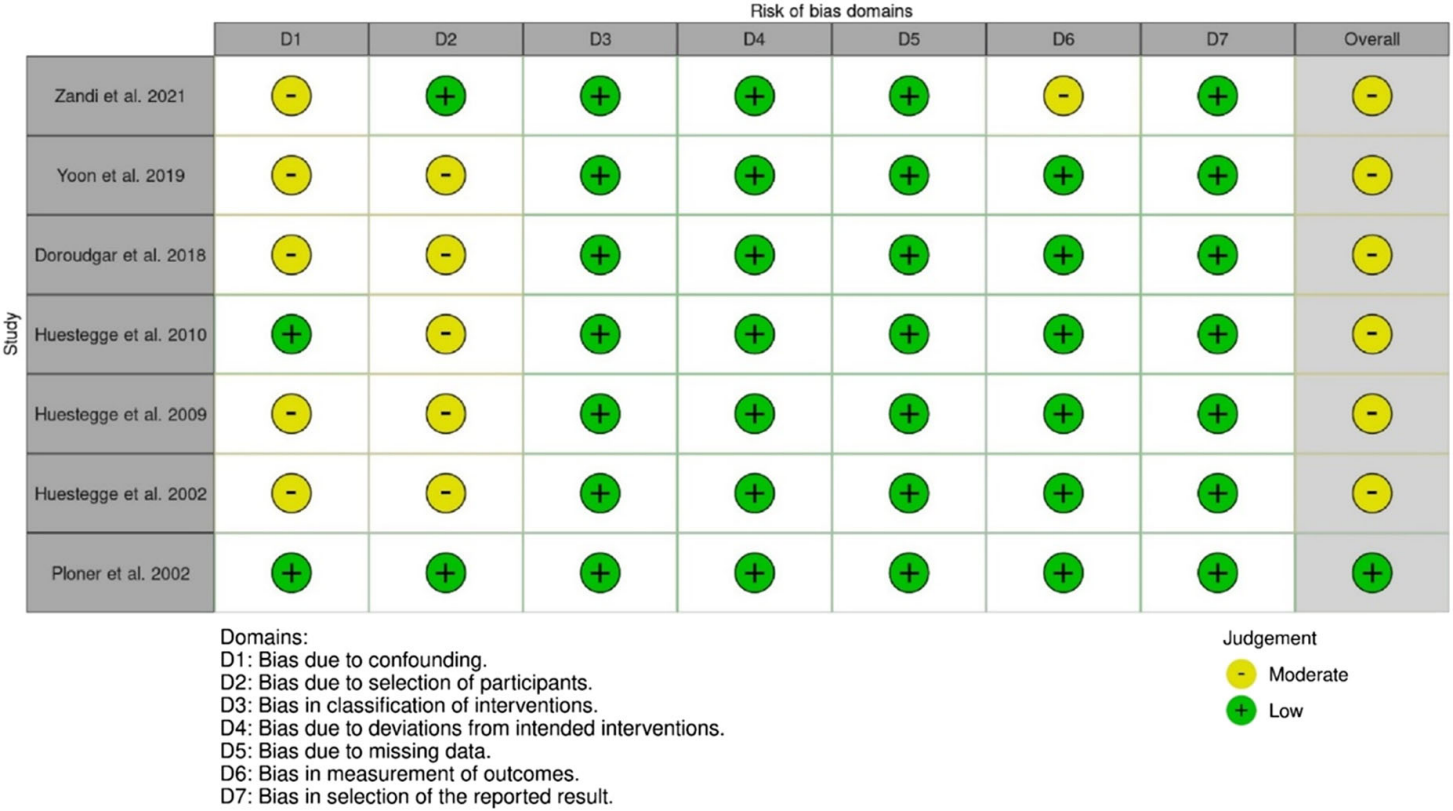
Domain and overall Cochrane Risk of Bias in Non‐Randomised Studies of Interventions (ROBINS‐I) judgements (reverse chronological order).

**FIGURE 5:**
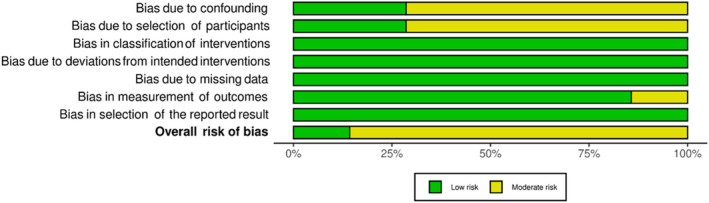
Domain and overall Cochrane Risk of Bias in Non‐Randomised Studies of Interventions (ROBINS‐I) judgement summary plot.

Nonrandomised studies had a low to moderate RoB (see Figures 4 and 5), with most at moderate risk due to confounding of the intervention effect and/or selection of participants. One study also had a moderate RoB due to the potential influence that participants' knowledge of the intervention received may have had on outcome measures.^39^ Only one study had a low RoB,^15^ while others were of some concern with a moderate RoB.^17^, ^26^, ^27^, ^35^, ^37^, ^39^

## 4 DISCUSSION

Driving is a complex and cognitively demanding skill that strongly relies on efficient and responsive oculomotor and visual attentional systems.^42^, ^43^, ^44^ This review offers a thorough assessment of cannabis/THC‐induced alterations to oculomotor control with consideration of how functional attention may be impacted during safety‐critical tasks such as driving. Cannabis consumption selectively alters specific ocular measures, such as saccadic latency and accuracy, visual scanning efficiency, HGN and smooth pursuit characteristics. These outcomes are discussed in turn below, within the context of a biometric framework for detecting impairment based on recognised standards of THC‐induced variations in neurobehaviour.^6^

### 4.1 Saccades

The effects of acute THC administration on saccadic latency appear selective, with some studies reporting increased latency,^15^, ^32^ while others do not observe this effect.^29^ Newer investigations have revealed that saccadic latencies tend to be prolonged in tasks that require immediate responses, but not in tasks where the response can be delayed, potentially indicating deficits in the timing mechanisms of saccade control.^26^ Consequently, observed increases in saccadic latency may be more associated with the initial phase of saccade programming, rather than response initiation or response execution at the motor level. Acute THC administration consistently decreased saccadic accuracy.^29^, ^32^ Decrements in accuracy may be linked to increased difficulty in withholding the premature execution of prepared saccades and in suppressing erroneous reflexive saccades in direct response to changes in visual cues.^15^ While our review did not explicitly examine visual attention deficits, reductions in saccadic accuracy may impact the continuity of visually derived attentional processes, aligning with findings that link decreased accuracy to poorer visual attention performance.^45^ THC did not reliably produce any variations in SPV or amplitude constants^15^, ^29^, ^30^, ^32^, ^35^ suggesting that while THC administration may selectively impact the timing and accuracy of saccades, it does not seem to affect the distance covered during these movements, indicating the preservation of basic brainstem related saccade generation.^26^ This observation is consistent with previous findings that oculomotor processes originating in cortical regions with low CB1 receptor density are less affected by cannabis use.^32^ Hence, it is likely that cannabis‐related deficits in neurocognition are not directly linked to saccade generation but rather may arise alongside neurobehavioural processes occurring further downstream.

Among CUD groups, similar mechanistic deficits were observed with greater antisaccade errors reflecting an overall visual inhibitory control deficit.^37^ A lack of prosaccade errors in the same group suggests no evidence of failures in instructional control nor global abnormalities in visual control.^37^ Deficits in saccade suppression and inhibitory control appear to manifest similarly across acute dosing scenarios and among chronic cannabis users. Increases in saccadic inaccuracy and latency reveal routine deficits in response selection, particularly in the ability to shift visual focus between target stimuli.^5^ In a driving scenario, this may functionally manifest as greater difficulty in attending to and processing changes in the environment, irrespective of potential tolerance to the effects of cannabis.

### 4.2 Gaze behaviour

Acute administration of THC did not reliably alter visual search frequency^38^ or other visual search characteristics^31^ among individuals with varied cannabis use. THC's inconsistent impact on visual search characteristics suggests that visual search behaviour may be more substantially affected by changes in task parameters and the surrounding environment. Cannabis has been shown to impair visual signal detection, yet greater signal detection failures were unrelated to oculomotor tracking performance (i.e. cannabis‐intoxicated individuals failed to recall what they were looking at despite successfully tracking the target), indicating potential working memory deficits.^46^ Cannabis‐related impairment of visual performance may not be impaired at central nervous system levels that control the ability of the eyes to track environmental stimuli but rather may be more dependent upon the nature and salience of the stimuli being attended to. This is problematic for tasks such as driving that are highly dependent on awareness of visual events, with changes in the drivers' dynamic visual field being left unattended to.

Acute administration of THC doses as low as 5 mg influenced temporal aspects of saccadic control during dynamic and demanding tasks such as driving, shown by an increase in fixation duration relative to placebo.^21^ Increases in fixation duration,^17^ rate and visual regressions^35^ were similarly shown in cannabis users who met diagnostic criteria for CUD with an early age of onset between 14 and 16 years old significantly, even in the absence of acute intoxication. Such results indicate that long‐term cannabis users with an early age of onset may be substantially less efficient in visual scanning with oculomotor control system impairments more enduring among CUD groups. These impairments further implicate deficits in working memory performance which have been repeatedly demonstrated in acute dosing cannabis studies,^47^ with THC intoxication linked to interferences in hippocampal and prefrontal activity during learning or tasks heavily reliant on memory.^45^ Seemingly, such deficits in working memory and oculomotor control appear to endure among chronic cannabis users with an early age of onset.

### 4.3 Eyelid characteristics

Acute THC administration did not impact blink duration, blink frequency, or eyelid opening in a pilot study^39^ but did reduce blink duration in a more rigorously controlled experimental study.^21^ Among individuals suspected of DUI, the presence of THC was associated with greater incidence of eyelid tremors compared with nondrug cases.^7^, ^8^, ^40^ Eyelid tremors demonstrate a potentially reliable biomarker in identifying cannabis‐related impairment, with good sensitivity (86%) and specificity (94%) among road users who have recently consumed cannabis.^7^ Recent evidence suggests that cannabis‐induced eyelid tremors may occur due to cannabinoid receptor's role in the modulation of ocular pain and inflammation that consequently triggers dry eye symptoms.^48^ While unlikely to be directly related to behavioural impairment, eyelid tremors may be a useful indicator of recent cannabis use and warrants further exploration.

### 4.4 Nystagmus

THC did not reliably produce VGN in experimental settings^34^, ^36^ nor among drivers suspected of DUI.^7^ Across studies utilising SFST to measure HGN, THC significantly increased impairment^27^, ^34^, ^36^ or trended towards impairment.^33^ Conversely, among studies utilising DEC tests, THC was not shown to produce any measurable effect on HGN.^7^, ^8^, ^40^ Divergent findings may be attributed to differences in study design, with SFST conducted under experimental settings while DEC tests were conducted solely in real‐world scenarios. Additionally, drivers apprehended at the roadside for suspected DUI may vary in their patterns of cannabis use compared with samples selected for more controlled clinical or observational studies.

In studies utilising DEC tests, an absence of effect may indicate differences in tolerance, with extant research indicating that more frequent cannabis use may protect from the impairing effects of THC on neurocognitive measures.^49^ Indeed, this may additionally offer support for Bosker and colleagues' findings,^33^ whereby an absence of significant effect of THC on HGN may be a sign of participants' ongoing chronic cannabis use, with additional acute administration producing little further impairment. As such, the sensitivity of roadside impairment tests in detecting HGN may be modulated by chronic cannabis use. Further investigation into the efficacy of field measures in identifying THC‐related impairment would benefit from a greater focus on how patterns of cannabis use may impact the sensitivity of such tests and assist in determining their suitability in accurately inferring driving‐relevant impairment.

### 4.5 Smooth pursuit

Reductions in smooth pursuit eye movement have been found to be an important indicator of the presence of HGN and has been significantly correlated to THC dosage.^36^ Studies which have moved beyond subjective reporting of nystagmus‐related cues to measure the effect of THC on smooth pursuit more objectively, however, have produced inconsistent results. Cumulative acute THC dosages produced no variations in smooth pursuit,^29^, ^32^ while decreased smooth pursuit^28^ and velocity^30^ were shown in comparable experimental designs, providing evidence of cannabinergic system involvement in some oculomotor processes related to motor control. Smooth pursuit reductions likely manifest as a shortfall in visual motion processing, with functional implications for drivers who may exhibit reduced accuracy in the tracking of moving targets, including other moving vehicles.^5^ Absence of robust impairment following THC administration may reflect the mediation of oculomotor control in widely distributed regions of the brain,^28^ with the sparsity of CB1 receptors found in lower brain stem areas that are responsible for some oculomotor functions potentially accounting for variations in smooth pursuit.^32^

### 4.6 Summary and limitations

Acute THC administration selectively impairs oculomotor control, increasing errors in saccade suppression and inhibitory control. These effects manifest as increased saccadic latency and inaccuracy, disrupting response selection and efficient processing of environmental changes, even occasionally in the absence of acute intoxication. Long‐term cannabis users, particularly those with an early onset of usage, often experience enduring oculomotor deficits, reducing visual scanning efficiency and increasing the likelihood that visual events go unattended to. The sensitivity of field sobriety and DEC tests in detecting HGN may vary with cannabis use chronicity, questioning the efficacy of such measures across diverse cannabis use populations. Smooth pursuit reductions, though inconsistent across studies, may suggest deficits in visual motion processing, potentially compromising the tracking of moving targets. Increased eyelid tremors are consistently observed with cannabis use; however, are unlikely to be directly linked with behavioural impairment. Future research would benefit from defining THC‐specific variations to oculomotor control, particularly along the broad spectrum of consumption patterns, to better inform the use of roadside impairment tests and implementation of ocular biomarkers in vehicle safety systems.

The current review should be considered in light of some practical limitations. The review's inclusion of diverse study designs and marked heterogeneity preclude methodological cross‐comparisons and the feasibility of meta‐analysis. Acute experimentally derived effects of cannabis products are not always concordant with residual effects of longer‐term cannabis use (particularly where recency and chronicity of use may remain unknown). Moreover, observational studies that did not necessitate participant abstinence from cannabis use may have inadvertently captured a mix of both acute and chronic effects. We also did not discriminate between studies based on the task employed during eye tracking, even though task complexity and environmental context likely play a role in oculomotor behaviour. Nonetheless, inclusion of varied study designs supports a more robust examination into relevant ocular parameters across diverse samples. In keeping these criteria broad, we provide a thorough revision of the literature in relation to various cannabis user groups and in doing so, expand the ecological relevance of our conclusions. We further limited the search criteria to cannabis administration or use inclusive of THC, subsequently excluding studies with cannabidiol only formulations or synthetic cannabinoids. While we acknowledge the growing popularity in the use of synthetic cannabinoids and their potential for harm, particularly among road users, it remains difficult to delineate synthetic cannabinoid findings in epidemiological research and restricts our ability to provide an authoritative commentary.

## 5 CONCLUSION

Acute THC consumption selectively impacts oculomotor control, notably increasing saccadic latency and inaccuracy while impairing inhibitory control. Long‐term cannabis users, especially those with early usage onset, display enduring oculomotor deficits that affect visual scanning efficiency. Further research is necessary to elucidate specific changes in oculomotor control induced by THC, which may enhance the precision of roadside impairment assessments and vehicle safety systems.

## AUTHOR CONTRIBUTIONS

**Brooke Manning**: Writing—original draft, methodology, data synthesisation, visualisation and writing—reviewing and editing. **Luke Downey:** Methodology, writing—reviewing and editing. **Andrea Narayan:** Data synthesisation, writing—reviewing and editing. **Amie Hayley:** Conceptualization, methodology, data synthesisation, visualisation, writing, original draft and writing—review & editing.

## CONFLICT OF INTEREST STATEMENT

BM, LD and AN have no conflicts of interests to declare.

## Supporting information


**Data S1.** Supporting Information.

## Data Availability

The authors confirm that the data supporting the findings of this study are available within the article and its supporting information.

## APPENDIX A RESULTS TABLES

**TABLE A1:** Study characteristics of included articles stratified by study design (reverse chronological order).

Authors, year	Study design	N subjects (% female)	Age, range, or *mean* years (±SD)	Cannabis use history
*Experimental*				
Manning et al (2023)	R, DB, PC, crossover	*N* = 14 (50)	21–58, 38.0 (10.78)	≥ 1 time in lifetime and ≤ 1 time per week
Shahadi Zandi et al (2021)	Pilot study, within subjects, baseline comparison	*N* = 10 (70)	19–26, 22.7 (2.79)	≥ 1 time per week and ≤ 4 times per week
Bosker et al (2012)	R, DB, PC, crossover	THC *N* = 20 (25); Baseline *N* = 19 (NR)	24.3 (1.4)	≥ 4 times per week in the last year
Kleinloog et al (2012)	R, DB, PC, crossover	*N* = 49 (0)	18–45, NR	≤ 1 time per week in the last year
Downey et al. (2011)	R, DB, PC, crossover	*N* = 80 (39)	21–35, 26.5 (5)	≥ 1 time in lifetime and ≤ 1 time per week
Liem‐Moolenaar et al. (2010)	R, DB, PC, partial crossover	THC *N* = 24 (0); Placebo *N* = 12 (0)	18–45, NR	≤ 1 time per week in the last year
Zuurman et al. (2008)	R, DB, PC, crossover	*N* = 12 (0)	21–27, 23 (2)	≤ 1 time per week in the last 6 months
Papafoutiou et al. (2005)	R, DB, PC, crossover	*N* = 40 (35)	21–35, 25.5 (3.1)	≥ 2 times per year in the last year and ≤ 1 time per week
Ploner et al. (2002)	Within subjects, baseline comparison	*N* = 12 (66)	24–31, 27.2 (NR)	≥ 1 time in lifetime, no regular use, and no use within last 4 weeks
Lamers & Ramaekers (2001)	R, DB, PC, crossover	*N* = 16 (50)	21–32, 23.6 (2.9)	≥ 1 time in the last month and ≤ 3 times per week
Fant et al. (1997)	FR, DB, PC, crossover	*N* = 10 (0)	24–31, 26.8 (NR)	≥ 2 times in the last month and ≤ 3 times per week
Moskowitz et al. (1976)	DB, PC, crossover	*N* = 9 (0)	21–26, 23.8 (NR)	≥ 1 time in lifetime and ≤ 3 times per week
*Observational*				
Porath & Beirness (2019)	Epidemiological	*N* = 721: THC cases = 541 (NR); Non‐drug cases = 180 (NR)	NR	NR
Yoon et al. (2019)	Cross‐sectional	*N* = 54: CUD group = 43 (40); Control group = 11 (45)	CUD group = 18–45, 29.8 (7.2); Control group = 18–45, 26.6 (4.7)	CUD group ≥ 4 times per week Control group < 10 times in lifetime
Doroudgar et al (2018)	Cross‐sectional	*N* = 72: CUD group = 31 (29); Control group = 41 (37)	CUD group = 19–65, 40.06 (NR); Control group = 23–71, 41.53 (NR)	CUD group ≥ 4 times per week; Control group had no cannabis use in the last year
Hartman et al. (2016)	Epidemiological	*N* = 584: THC cases = 302 (13); Non‐drug cases = 282 (11)	THC cases = 15–59, 21 (med); Non‐drug cases = 15–59, 34 (med)	NR
Porath‐Waller & Beirness (2014)	Epidemiological	*N* = 843: THC cases = 703 (NR); Non‐drug cases = 140 (NR)	NR	NR
Huestegge et al (2010)	Cross‐sectional	*N* = 40: CUD group = 20 (30); Control group = 20 (30)	CUD group = 19–45, 25 (NR); Control group = 19–45, 24 (NR)	CUD group ≥ 2 times per week in the last 2 years Early onset 14–16 years; Control group had no past cannabis use
Huestegge et al (2009)	Cross‐sectional	*N* = 40: CUD group = 20 (30); Control group = 20 (30)	CUD group = 19–45, 25 (NR); Control group = 19–45, 24 (NR)	CUD group ≥ 2 times per week in the last 2 years Early onset 14–16 years; Control group had no past cannabis use
Huestegge et al (2002)	Cross‐sectional	*N* = 37: CUD group = 17 (18); Control group = 20 (NR)	CUD group = 19–45, 24.9 (7.8); Control group = NR	CUD group ≥ 3 times per week in the last 4 years Early onset 14–16 years; Control group had no past cannabis use

Abbreviations: CUD, cannabis use disorder; DB, double blinded; FR, force randomised; med, median; NR, not reported; PC, placebo controlled; R, randomised.

**TABLE A2:** Summary of the effect of cannabis/delta‐9 tetrahydrocannabinol (THC) on oculomotor outcomes, stratified by study design (reverse chronological order).

Author, year	THC consumption (administration route, dose, cannabis use frequency)	Outcome (measurement, single/multiple assessment, time post consumption)	Results	Summary
*Experimental randomised controlled trials and pilot studies (acute administration)*				
Manning et al. (2023)	Oral solution ‐ THC 5 mg Light ‐ moderate cannabis use history (abstained for >2 weeks prior to study)	Fixation and blink duration/rate (mean): SensoMotoric Instruments eyetracker (head‐mounted infrared camera with sampling rate of 50 Hz) Single eye tracking recording (100‐140mins)	Gaze behaviour: Fixation duration increased^↓^ ^a^, * ↔ Fixation rate^a^ Eyelid characteristics: Blink duration decreased^a^, * ↔ Blink rate^a^	Concurrent increases in fixation duration and reductions in blink duration after THC consumption support the role of cannabis in the regulation of oculomotor control. These findings also implicate the cannabinergic system in influencing the temporal aspects of saccadic control.
Shahidi Zandi et al. (2021)	Inhaled (cigarette) ‐ THC (mean) 62.72 mg (19.15 mg) Light – heavy cannabis use history (abstinence NR)	Saccade velocity, gaze behaviour (standard deviation of pitch angle), and blink duration/rate: SmartEye Pro 8.0 (two infrared cameras with sampling rate of 60 Hz) Multiple eye tracking recordings (15min, 35min, 1 h, 2 h, 3 h, 4 h, and 5 h)	Saccades: Decreased saccade velocity during 5 sec epochs associated with increases in blood THC concentration^b^ Gaze behaviour: Increased gaze pitch angle associated with increases in blood THC concentration^b^ Eyelid characteristics: ↔ Blink/eyelid closure features^b^	Increases in blood THC concentration were correlated with slower saccadic velocity and greater gaze pitch.
Bosker et al. (2012)	Inhaled (cigarette) ‐ THC 400 μg/kg Heavy cannabis use history (ongoing)	SFST ‐ HGN^2^ Single SFST assessment (2 h)	Nystagmus: HGN impairment increased (trend) ^↓^ ^b^	THC increased errors in HGN, but this change only approached significance. Lack of significance may reflect THC presence at baseline, as no separate control group was utilised.
Kleinloog et al. (2012)	Inhaled (vapour) ‐ THC 2 mg, 4 mg, 6 mg consecutively Light – moderate cannabis use history (negative THC drug test prior to study)	Saccades: Stimulated at an amplitude of approximately 15° to either side, with interstimulus intervals varying randomly between 3–6 s^1^ Smooth pursuit (% of time of which eye movements are in smooth pursuit of the target): Stimulated in a sinusoidal manner at frequencies ranging from 0.3–1.1 Hz and amplitude of 22.5° to either side^1^ Multiple eye tracking recordings after each consecutive dose (NR)	Saccades: Saccadic inaccuracy increased ^↓^ ^a^, * ↔ SPV^a^ ↔ Saccadic reaction time^a^ Smooth pursuit: ↔ Smooth pursuit eye movement^a^	THC produced a slight decrease in visual attention as measured by saccadic inaccuracy.
Downey et al. (2011)	Inhaled (cigarette) – THC 1.8% (0.81 g), THC 3% (1.78 g) Light – moderate cannabis use history (negative THC drug test prior to study)	Standardised Field Sobriety Tests (SFST) – Horizontal gaze nystagmus (HGN)^2^ SFST – Vertical gaze nystagmus (VGN)^3^ Single SFST assessment (50min)	Nystagmus: HGN impairment increased^↓^ ^a^, * ↔ VGN^a^	At both dosages, THC produced greater errors in HGN but not in VGN.
Liem‐Moolenaar et al. (2010)	Inhaled (vapour) ‐ THC 2 mg, 4 mg, 6 mg consecutively Light – moderate cannabis use history (negative THC drug test prior to study)	Average values of SPV (deg/s): Stimulated at amplitudes of 15 to 20° to either side, with interstimulus intervals varying randomly between 4–8 s^1^ Smooth pursuit (% of time of which eye movements are in smooth pursuit of the target): Stimulated in a sinusoidal manner with frequencies ranging from 0.3–1.1 Hz and amplitude of 20° to either side^1^ Multiple eye tracking recordings: post first dose (3 h‐4h15m), second dose (4h30m‐5h35m), and third dose (6 h‐7h10m)	Saccades: ↔ SPV^a^ Smooth pursuit: Smooth pursuit eye movement decreased^↓^ ^a^, *	THC produced a decrease in smooth pursuit eye movements, providing evidence for the role of cannabinoid systems in some oculomotor processes related to motor control.
Zuurman et al. (2008)	Inhaled (vapour) ‐ THC 2 mg, 4 mg, 6 mg, 8 mg consecutively Light – moderate cannabis use history (negative THC drug test prior to study)	Saccadic inaccuracy (%); Average values of SPV (deg/s); Saccadic reaction time (Ms): Stimulated at an amplitude of approximately 15° to either side, with interstimulus intervals varying randomly between 3–6 s^1^ Smooth pursuit (% of time of which eye movements are in smooth pursuit of the target): Stimulated in a sinusoidal manner at frequencies ranging from 0.3–1.1 Hz and amplitude of 22.5° to either side^1^ Multiple eye tracking recordings after each consecutive dose (NR)	Saccades: After fourth consecutive dose: Saccadic latency increased^↓^ ^a^, * Saccadic inaccuracy increased^↓^ ^a^, * ↔ SPV^a^ Smooth pursuit: ↔ Smooth pursuit eye movement^a^	At the highest cumulative dose, THC only produced changes to saccadic latency and saccadic accuracy. CB1 receptors sparsely found in lower brain stem areas responsible for some oculomotor functions may explain why few changes in SPV and smooth pursuit eye movement were seen.
Papafoutiou et al. (2005)	Inhaled (cigarette) ‐ THC 1.74% (0.813 g), THC 2.93% (1.776 g) Light – moderate cannabis use history (negative THC drug test prior to study)	SFST – HGN^2^ SFST – VGN^3^ Multiple SFST assessments (5 min, 55min, and 105min)	Nystagmus: HGN impairment increased at 55‐mins** and 105‐mins* after smoking^↓^ ^a^ ↔VGN^a^	THC produced greater errors in HGN at 55 min and 105 min but not at 5 min, suggesting that HGN impairment may manifest during the elimination phase in which ‘dumped’ THC re‐enters the blood stream.
Ploner et al. (2002)	Oral solution ‐ THC 10 mg Light cannabis use history (abstained for >4 weeks prior to study)	Saccade latency, saccadic peak velocity (SPV), saccade accuracy, saccade amplitude (constants), and anticipatory saccades: Eyetracker using horizontal infrared oculography of the right eye with a sampling rate of 200 Hz Single eye tracking recording (2 h)	Visually guided saccades: Latency increased^↓^ ^b^, ** ↔ SPV^b^ ↔ Amplitude^b^ ↔ Accuracy^b^ Memory‐guided saccades: Anticipatory saccades increased^↓^ ^b^, * Accuracy decreased ^↓^ ^b^, * ↔ SPV^b^ ↔ Amplitude^b^ Antisaccades: Antisaccade errors increased^↓^ ^b^, **	THC selectively impacted several parameters of saccadic eye movement suggesting the modulation of neuronal substrates of the network controlling saccades. These results provide evidence for the involvement of the cannabinoidergic system in the control of saccadic eye movements.
Lamers & Ramaekers (2001)	Inhaled (cigarette) ‐ THC 100 μg/kg Light – moderate cannabis use history (negative THC drug test prior to testing)	Visual search behaviour: 4000SU Eye Tracker head‐mounted eye tracking system Single eye tracking recording (25 min)	Gaze behaviour: ↔ Visual search frequency^a^	There were no significant THC effects on visual search frequency at traffic intersections. Absence of effect may reflect relatively low THC dosage.
Fant et al. (1997)	Inhaled (cigarette) ‐ THC 15.6 mg, THC 25.1 mg Moderate cannabis use history (abstinence NR)	Smooth pursuit (deg/s): Eye Performance System 100 tests for smooth pursuit movement in each eye as subjects track a target moving between 0 and 45° of horizontal visual angle Multiple eye tracking recordings (15 m, 1 h, 1 h 45 min, 2 h 30 m, and 3 h 30 m)	Smooth pursuit: Smooth pursuit velocity decreased^↓^ ^a^, * at THC (25.1 mg)	At the highest dose, THC produced consistent decrements in smooth pursuit eye tracking in the central (0 to 22 deg) and peripheral (22 to 45 deg) visual fields. These results provide evidence for the mediation of oculomotor control in widely distributed regions of the brain.
Moskowitz et al. (1976)	Inhaled (cigarette) ‐ THC 200 μg/kg Light – heavy cannabis use history (abstinence NR)	Gaze behaviour: Eye Point of Regard head‐mounted eye tracking system Single eye tracking recording (25 min)	Gaze behaviour: ↔ Visual search characteristics^a^	There were no significant THC effects on visual search characteristics. Absence of effect may expose the sensitivity of visual search behaviour to factors of set/nature of stimuli being attended to.
*Cross‐sectional studies (chronic/ongoing cannabis use)*				
Yoon et al. (2019)	Varied administration route and THC dosage Heavy cannabis use history (ongoing)	Prosaccades and antisaccades (no. of errors): MiraMetrix S2 Eyetracker with sampling rate of 60 Hz	Saccades: Antisaccade errors increased^↓^ ^c^, *** ↔ Prosaccade errors^c^	The CUD group produced greater antisaccade errors, reflecting an overall visual inhibitory control deficit. However, lack of prosaccade errors suggests no evidence of failures in instructional control nor global abnormalities in visual control.
Doroudgar et al. (2018)	Varied administration route and THC dosage Heavy cannabis use history (ongoing)	SFST ‐ HGN^2^	Nystagmus: HGN impairment increased^↓^ ^c^ ***	Greater errors in HGN were apparent in heavy/chronic cannabis users compared with non‐users.
Huestegge et al. (2010)	Inhaled (cigarette) – varied THC dosage Heavy cannabis use history (abstained for >24 h prior to testing) and early onset (14–16 years old)	Saccadic amplitude, fixation duration (mean), and visual regression; EyeLink infrared head‐mounted eye tracking system with a sampling rate of 250 Hz	Saccades: ↔ Saccade amplitude^c^ Gaze behaviour: Fixation duration increased^↓^ ^c^, * Visual regressions increased^↓^ ^c^, *	These results suggest that long‐term deficits of the oculomotor control system may be present among CUD groups as characterised by general slowing of saccade programming in complex tasks and greater deficits in working memory performance.
Huestegge et al. (2009)	Inhaled (cigarette) – varied THC dosage Heavy cannabis use history (abstained for >24 h prior to testing) and early onset (14–16 years old)	Saccades latency (mean), initial saccade amplitude (mean), initial saccade latency (mean), and prosaccade errors: EyeLink infrared head‐mounted eye tracking system with a sampling rate of 250 Hz	Prosaccades: Saccade latency increased^↓^ ^c^, * Initial saccade latency decreased^c^, ** ↔ Initial saccade amplitude^c^ ↔ Prosaccade errors^c^ Antisaccades: Saccade latency increased^↓^ ^c^, * Initial saccade latency decreased^c^, ** Initial saccade amplitude increased^↓^ ^c^, ** Memory‐guided saccades: Saccade amplitude increased at 3° eccentricity^c^, * ↔ Saccade latency^c^ ↔ Saccade amplitude at 6° eccentricity^c^	Saccadic latencies are present in immediate response tasks but absent in delayed response tasks, suggesting potential deficits in temporal aspects of saccade control. Such deficits may be associated with the initial phase of saccade programming rather than due to the process of response initiation or response execution at the motor level.
Huestegge et al. (2002)	Inhaled (cigarette) – varied THC dosage Heavy cannabis use history (abstained for >16 h prior to testing) and early onset (14–16 years old)	Saccade amplitude (deg/s), average values of SPV (deg/s), gaze/scan paths (matrix): EyeLink infrared head‐mounted eye tracking system with a sampling rate of 250 Hz	Saccades: Saccadic amplitude increased^↓^ ^c^, * ↔ SPV^c^ Gaze behaviour: Fixations per item (mean) increased ^↓^ ^c^, * Visual regressions increased ^↓^ ^c^, * ↔ Fixation duration^c^	Results indicate that long‐term cannabis users with early age of onset are substantially less efficient in visual scanning.
*Epidemiological studies (roadside drug testing)*				
Porath & Beirness (2019)	Unknown	Drug Evaluation and Classification (DEC) tests ‐ HGN^2^ Eyelid tremors^4^	Nystagmus: ↔ HGN impairment^d^ Eyelid characteristics: Eyelid tremors increased^↓^ ^d^, **	Across studies utilising DEC tests, THC alone did not product a measurable effect on HGN or VGN. Absence of effect may reflect differences in tolerance to the impairing effects of THC as a result of differences in cannabis use history. All three studies showed an increased proportion of participants displaying eyelid tremors after consumption of THC. These results provide further support for the active involvement of the receptor TRPA1 in sensory neurons of the peripheral cannabinoid pathway.
Hartman et al. (2016)	Unknown	DEC tests – HGN^2^ and VGN^3^ Eyelid tremors^4^	Nystagmus: ↔ HGN impairment^d^ ↔ VGN impairment^d^ Eyelid characteristics: Eyelid tremors increased^↓^ ^d^, *	
Porath‐Waller & Beirness (2014)	Unknown	DEC tests ‐ HGN^2^ Eyelid tremors^4^	Nystagmus: ↔ HGN impairment^d^ Eyelid characteristics: Eyelid tremors increased^↓^ ^d^, *	

*Note*: ↑ = improved performance; ↓ = impaired performance; ↔ = no significant difference in performance. NR = not reported. Light cannabis use (≥ 1 time in lifetime and ≤1 time per month), moderate cannabis use (≥ 2 times per month and ≤ 9 times per month), heavy cannabis use or cannabis use disorder (CUD) populations (≥ 10 times per month or met the DSM‐V diagnosis criteria for CUD).

^a^ THC vs placebo.

^b^ THC vs baseline.

^c^ CUD vs control group.

^d^ THC cases vs non‐drug group.

^1^ Electrodes placed on the forehead and next to both lateral canthi.

^2^ Presence or absence of (1) lack of smooth pursuit, (2) distinct nystagmus at maximum deviation, (3) nystagmus onset before 45°.

^3^ Nystagmus onset at maximum deviation in an upward vertical gaze.

^4^ Presence/absence of eyelid tremors during DEC tests.

* *p* < 0.05.

** *p* < 0.01.

*** *p* < 0.001.
