# Supplementary material for: A systematic review of oculomotor deficits associated with acute and chronic cannabis use
Source: Addict Biol. 2023 Dec 19;29(1):e13359. doi: 10.1111/adb.13359 (PMC10898834; doi:10.1111/adb.13359)
Supplement: Supplementary file 1 — Data S1. Supporting Information. [file ADB-29-e13359-s001.docx]

**Supplementary: Risk of bias justification**

**Table 3**

*Justification for Cochrane Risk of Bias (RoB 2) assessments for included randomised* *crossover studies:*

| Author | Year | Overall Rating | Domain | Rating | Justification |
| --- | --- | --- | --- | --- | --- |
| Bosker et al. | 2012 | S | D1 | S | No information reported on whether the allocation sequence was concealed until participants were enrolled and assigned to interventions. |
| Fant et al. | 1997 | H | D1;  DS;  D5 | H;  H;  S | The allocation sequence was not fully concealed until participants were enrolled and assigned to interventions due to forced randomisation; insufficient time for any carryover effects (≤ 4 days) to have disappeared before outcome assessments in following periods; unable to find statistical analysis plan that produced the result analysed. |
| Kleinloog et al. | 2012 | S | D5 | S | Unable to find statistical analysis plan that produced the result analysed. |
| Lamers & Ramaekers | 2001 | S | D5 | S | Unable to find statistical analysis plan that produced the result analysed. |
| Moskowitz et al. | 1976 | H | D1;  D5 | H;  S | No information reported on randomization or whether the allocation sequence was concealed until participants were enrolled and assigned to interventions; unable to find statistical analysis plan that produced the result analysed. |
| Papafoutiou et al. | 2005 | S | D5 | S | Unable to find statistical analysis plan that produced the result analysed. |
| Zuurman et al. | 2008 | S | D5 | S | Unable to find statistical analysis plan that produced the result analysed. |

*Note:* H = High risk of bias; S = Some concerns

**Table 4**

*Justification for Cochrane Risk of Bias in Non-Randomised Studies of Interventions (ROBINS-I) assessments:*

| Author | Year | Overall Rating | Domain | Rating | Justification |
| --- | --- | --- | --- | --- | --- |
| Doroudgar et al. | 2018 | M | D1;  D2 | M;  M | Cannabis use age of onset, cannabis use disorder severity, and ethnicity may result in the potential for confounding of the effect of cannabis use, with appropriate analysis (*t* Tests, Chi-square, and Fisher’s exact tests); start of follow-up and start of intervention do not coincide as prevalent rather than new users of cannabis were recruited, without adjustment techniques. |
| Huestegge et al. | 2002 | M | D1;  D2 | M;  M | Recent cannabis use and cannabis use disorder severity may result in the potential for confounding of the effect of cannabis use, with appropriate analysis (ANOVA); start of follow-up and start of intervention do not coincide as prevalent rather than new users of cannabis were recruited, without adjustment techniques. |
| Huestegge et al. | 2009 | M | D1;  D2 | M;  M | Recent cannabis use and cannabis use disorder severity may result in the potential for confounding of the effect of cannabis use, with appropriate analysis (multi-factor ANOVA); start of follow-up and start of intervention do not coincide as prevalent rather than new users of cannabis were recruited, without adjustment techniques. |
| Huestegge et al. | 2010 | M | D1;  D2 | M;  M | Recent cannabis use and cannabis use disorder severity may result in the potential for confounding of the effect of cannabis use with appropriate analysis (*t* Tests and mixed ANOVA); start of follow-up and start of intervention do not coincide as prevalent rather than new users of cannabis were recruited, without adjustment techniques. |
| Yoon et al. | 2019 | M | D1;  D2 | M;  M | Cannabis use age of onset, age, gender, and years of education may result in the potential for confounding of the effect of cannabis use, with appropriate analysis (regression and Wilcoxon signed-rank test); start of follow-up and start of intervention do not coincide as prevalent rather than new users of cannabis were recruited, without adjustment techniques. |
| Zandi et al. | 2021 | M | D1;  D6 | M;  M | Cannabis use history and age of onset may result in the potential for confounding of the effect of cannabis use, with appropriate analysis (regression and ANOVA); participants’ knowledge of the intervention received may have influenced outcome measures. |

*Note:* M=Moderate risk of bias
